# Analysis of Depth Cameras for Proximal Sensing of Grapes

**DOI:** 10.3390/s22114179

**Published:** 2022-05-31

**Authors:** Baden Parr, Mathew Legg, Fakhrul Alam

**Affiliations:** Department of Mechanical and Electrical Engineering, Massey University, Auckland 0632, New Zealand; 1badenparr@gmail.com (B.P.); f.alam@massey.ac.nz (F.A.)

**Keywords:** grapes, yield estimation, depth cameras, RGB-D

## Abstract

This work investigates the performance of five depth cameras in relation to their potential for grape yield estimation. The technologies used by these cameras include structured light (Kinect V1), active infrared stereoscopy (RealSense D415), time of flight (Kinect V2 and Kinect Azure), and LiDAR (Intel L515). To evaluate their suitability for grape yield estimation, a range of factors were investigated including their performance in and out of direct sunlight, their ability to accurately measure the shape of the grapes, and their potential to facilitate counting and sizing of individual berries. The depth cameras’ performance was benchmarked using high-resolution photogrammetry scans. All the cameras except the Kinect V1 were able to operate in direct sunlight. Indoors, the RealSense D415 camera provided the most accurate depth scans of grape bunches, with a 2 mm average depth error relative to photogrammetric scans. However, its performance was reduced in direct sunlight. The time of flight and LiDAR cameras provided depth scans of grapes that had about an 8 mm depth bias. Furthermore, the individual berries manifested in the scans as pointed shape distortions. This led to an underestimation of berry sizes when applying the RANSAC sphere fitting but may help with the detection of individual berries with more advanced algorithms. Applying an opaque coating to the surface of the grapes reduced the observed distance bias and shape distortion. This indicated that these are likely caused by the cameras’ transmitted light experiencing diffused scattering within the grapes. More work is needed to investigate if this distortion can be used for enhanced measurement of grape properties such as ripeness and berry size.

## 1. Introduction

Accurate and timely yield estimation can have a significant effect on the profitability of vineyards. Among other reasons, this can be due to better management of vineyard logistics, precise application of vine inputs, and the delineation of grape quality at harvest to optimise returns. Traditionally, the process of yield estimation is conducted manually. However, this is destructive, labour-intensive and time-consuming leading to low sampling rates and subjective estimations [1]. Automating yield estimation is therefore the focus of ongoing research in the computer vision field [2].

Current 2D camera techniques predominantly rely on distinct features of grapes, such as colour or texture, to identify and count individual berries within RGB (Red, Green, and Blue) images [3,4]. However, the accuracy of yield estimations from these approaches is greatly restricted by the proportion of grapes visible to the camera. Hence, occlusion of grapes is an issue. Additionally, errors in the sizing of grapes can occur unless the distance between the camera and the grapes is known.

An alternative technique, which has been reported to provide improved yield accuracy, has been to incorporate 3D information. Grape bunch 3D architectonic modelling has been performed from high-resolution 3D scans of grape bunches within lab environments. These have been achieved using commercial laser scanners [5,6] and blue LED structured light scanners [7,8,9,10]. These scans can be used to estimate volume, mass, and number of berries per bunch. However, these 3D scanners are costly, require significant time to capture viable point clouds, and their use is yet to be demonstrated within field environments.

High-resolution 3D scans of grapes and vines have also been achieved using multiple RGB images captured from different positions using structure from motion photogrammetry techniques [11,12,13]. This method can be used with inexpensive equipment [14] and data collection can be automated by mounting cameras on platforms such as robots or drones [15]. However, generating photogrammetry scans requires significant computation load and time. Rose et al. [12] quoted 8 h to generate a point cloud for one 25 m length of vine.

An alternative approach that has been investigated is to identify within an RGB image the location and size of individual berries within a bunch and use this information to model the 3D grape bunch architecture using spheres or ellipsoid shapes. Liu et al. [16,17,18,19] used a backing board behind the grape bunch when capturing the RGB images to aid with the segmentation of individual berries. Berry size was estimated by placing a chequerboard pattern on the board. This allowed the distance between the camera and the backing board to be measured using camera calibration techniques. However, this requirement for a backing board means it can only be used for handheld applications. Ivorra et al. demonstrated/developed a novel technique that utilised a stereoscopic RGB-D (Red, Green, Blue—Depth) camera to obtain berry size without having to use a chequerboard pattern. They combined the depth information with 2D image analysis to achieve 3D modelling of the grape bunches.

The potential real-time benefits of RGB-D cameras for grape yield estimation have encouraged researchers to investigate their use for grape yield estimation. A range of low-cost RGB-D cameras that can generate 3D scans in real-time has become available on the market in recent years. This has been driven by their use in a wide range of applications including gaming, robotics, and agriculture. The main technologies used are stereoscopy, Active Infrared Stereoscopy (AIRS), Structured Light (SL), Time of Flight (ToF), and Light Detection And Ranging (LiDAR). Stereoscopy is similar to human vision and uses parallax and disparity between featured in images from cameras that are spatially separated. Active infrared stereoscopy is similar but projects an Infrared (IR) pattern into the scene to assist with finding correspondences. This is particularly useful for cases where objects being scanned have low visible texture and/or are in low light conditions. Structured light detects distortions in a known projected IR pattern. Time of flight and LiDAR cameras both operate by measuring the time taken for emitted IR light to be reflected back to the camera. ToF cameras typically emit this light in a single pulse, while LiDARs typically measure by sweeping a laser. RGB-D cameras have been used for 3D imaging a range of different fruits [20]. This includes several studies related to imaging grapes.

Marinello et al. [21] used a Kinect Version 1 (V1) camera, which operates using IR structured light, to image grapes in a lab environment for yield estimation. Their results showed that the scanning resolution decreased significantly with the increased distance of the sensor from the grapes. Hacking et al. [22,23] also used the Kinect V1 for yield estimation in both lab and vineyard environments. They showed that the Kinect V1 gave a good correlation with grape bunch volume in the lab but struggle in the field environment. They suggested that this could be due to sunlight and the presence of leaves. They recommended that future work should investigate the performance of the Kinect V2, since it is a ToF camera and hence is more robust to sunlight conditions compared with SL cameras, such as the Kinect V1, which project IR patterns [24]. An alternative approach could be to take measurements at night. This technique has been used by studies capturing traditional RGB images in vineyards [3,25].

Kurtser et al. [26] used an Intel RealSense D435 RGB-D camera, which operates using AIRS technology, for imaging grapes bunches in an outdoor environment. They used neural networks for detecting grape bunches from the point clouds [27]. Basic shapes (box, ellipsoid, and cylinder) were fitted to the point clouds. However, they reported relatively large (28–35 mm) errors in the length and width of these fitted shapes compared with the physical measurement of the grape bunches. These errors were reported to be affected by sunlight exposure. It would appear that in sunlight conditions, the projected IR pattern would not be viable meaning this camera would be acting as a stereo camera.

Ivorra et al. [28] used a stereoscopic RGB-D camera (Point Grey Bumblebee2) for imaging grapes, as mentioned above. However, the 3D scans of the grapes from this camera were of poor quality. They suggested that this was due to difficulty in making the correct correspondence between the stereo image pairs. Yin et al. [29] also used a stereoscopic camera (ZED) for imaging grapes. However, this was used to measure the pose of grape bunches for automated robotic picking rather than yield estimation.

This article presents the first benchmarking of the performance of multiple RGB-D cameras for use in grape yield estimation applications. This includes ToF cameras, which have not been used before in a grape yield estimation study. The benchmarking performance analysis was obtained by calculating error maps between high-resolution scans obtained using photogrammetry and those obtained by the RGB-D cameras. This includes an analysis of the cameras’ performance in and out of direct sunlight.

Previous studies [21,22,23,26,27,28] have only looked at volume errors for a grape bunch as a whole. However, in this work, depth map errors in the RGB-D scans of grapes are analysed at an individual grape berry scale, which has not been done before.

The ability to identify individual grapes from 3D scans would provide additional information for the yield and crop load estimation process. This could inform viticulturists of metrics such as berry size distribution and berry count per cluster. There is also the potential for more accurate volume estimates by 3D modelling of the grape cluster architecture. This has been explored by several researchers [5,6,7,8,9,10,16,17,18,19,28] but not for RGB-D cameras. This might be because it has been thought that these cameras did not have sufficient precision [5].

In this work, the ability of RGB-D cameras for detecting individual grape berries using Random Sample Consensus (RANSAC) is investigated. We are not aware of any reported works that have applied an algorithm such as RANSC with RGB-D camera scans for grape berry detection.

The remainder of the article is organised as follows. Section 2 describes the experimental setup and data processing used. The results are presented in Section 3. Section 4 provides a discussion on the results. Finally, a conclusion is provided in Section 5.

## 2. Methodology

### 2.1. Hardware and Measurement Procedure

The RGB-D cameras used in this work were chosen to cover the main technologies available. The cameras used were the Kinect V1 (SL), Intel RealSense D415 (AIRS), Microsoft Kinect V2 (ToF), Microsoft Kinect Azure (ToF), and Intel L515 (LiDAR). Table 1 provides some specifications on these cameras. Additionally, a Sony Alpha A6300 mirrorless RGB camera was used to obtain high-resolution scans of the grapes using photogrammetry. Note that the Kinect V1 and Kinect V2 are discontinued. However, the Kinect V2 is still very commonly used in research and both are used or mentioned in the related literature. Including the results from these two cameras also provides benchmarking of the newer with older camera technologies.

**Figure 1:**
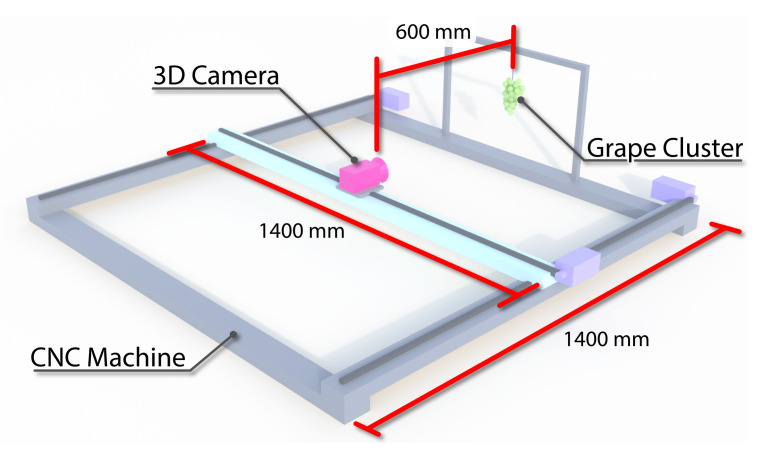
Diagram showing setup of camera and grapes mounted onto the CNC machine for capturing RGB-D images.

The RGB-D cameras were mounted on a 2D gantry (CNC machine). The gantry had a 2D travel range of 1400 × 1400 mm and a resolution of 0.025 mm. A bunch of green table grapes was suspended in front of the cameras at one end of the gantry. The gantry system was used to move the camera under investigation directly in front of and at the desired distance from the grapes, see Figure 1.

**Figure 2:**
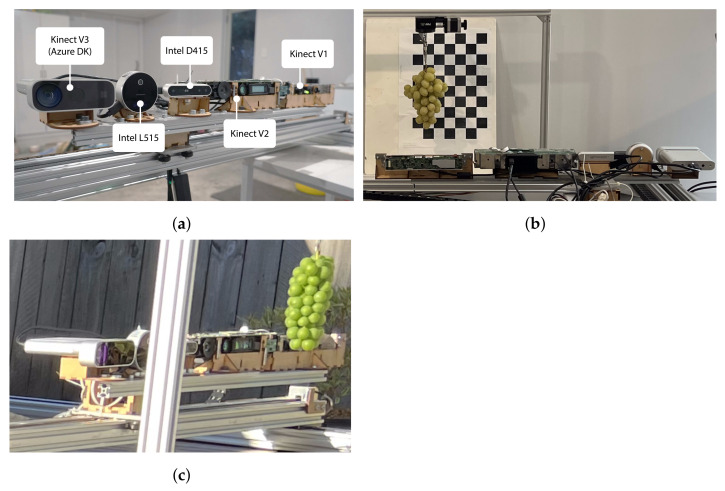
Photos of the experimental setup. Photo (**a**) shows the front view of the cameras mounted onto the 2D gantry. Photos (**b**,**c**) respectively show the setup located inside and outdoors.

**Figure 3:**
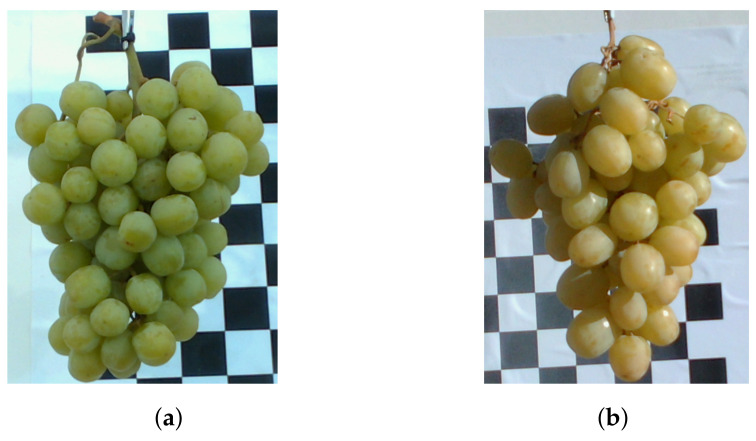
Coloured images of the grape bunches used in this work for scans captured (**a**) indoors and (**b**) outdoors.

Figure 2 provides photos of the experimental setup. Figure 3 shows photos of the grapes used in this work for both indoor and outdoor measurements. These are cropped versions of the images captured by the Intel L515 camera, which was located 600 mm from the grapes.

Python code was used to move the gantry so that a camera under investigation was directly in front of the grape bunch and then capture RGB-D images with the camera at a number of distances from the grapes. Most of the measurements shown in this work were with the camera located at a distance of 600 mm from the grapes. This distance was used as it was a distance that worked well for all cameras tested. For example, the Kinect V1 and V2 struggled to capture images at distances much closer than this. The newer cameras were able to image at closer ranges. In addition to this, it was felt that this distance was likely to be a practical separation distance of the cameras from the grapes if the camera was mounted onto a farm vehicle driving between vine rows. This process was then repeated for all the RGB-D cameras. The Sony Alpha A6300 mirrorless RGB camera was then used to capture RGB (6000 × 4000 pixel) images of the grapes at a range of positions for high-resolution photogrammetry scans. The above measurement process was performed first in the lab and then outdoors in direct sunlight using a different grape bunch. This was done to evaluate the effect of sunlight on the performance of each RGB-D camera.

**Figure 4:**
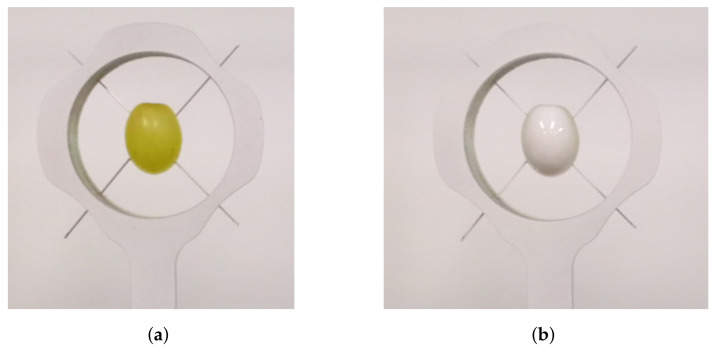
Photos of the setup of scans for a single grape which is first unpainted (**a**) and then painted (**b**). This was performed to analyse the effect of diffused scattering within the grape for the RGB-D cameras, which use ToF and LiDAR technologies.

Measurements were also performed to evaluate if diffused scattering within the grapes was causing distortions in the ToF and LiDAR cameras. This was achieved by obtaining scans before and after spraying the grapes with white primer paint. The paint aimed to make the grapes opaque and hence stop diffused scattering within the berries. Figure 4 shows the setup used for a single grape positioned inside a ring before and after it has been sprayed with paint. Needles were used to secure the grape and ensure that the front face of the grape was flush with the front surface of the ring. Care was taken to not pierce the grape so as not to disrupt the internal optics of the grape.

### 2.2. Processing Data

The software Agisoft Metashape v1.5.2 was used to obtain high-resolution photogrammetry scans of the grape bunches using the RGB images captured by the Sony A6300 from a number of positions. These provided a baseline scan that could be used to evaluate the accuracy of the RGB-D cameras. The point clouds obtained using both the RGB-D and photogrammetry scans were then processed using CloudCompare. This is a widely used 3D point cloud and mesh processing open-source software. It has a range of point cloud processing tools including cropping, filtering, alignment, distance measurement, and comparison of multiple point clouds.

It was observed that the raw ToF and LiDAR camera scans had a significant number of flying pixels around the edges of the grape bunch. A significant portion of these was therefore filtered out using CloudCompare. This was done by rejecting points that had normal angles greater than a set value. This was empirically chosen to be 85 degrees. Isolated points were then discarded using statistical outlier rejection, which compared distances between its six nearest neighbours and used one standard deviation of the entire point cloud distribution as the rejection threshold [30].

#### 2.2.1. Alignment of Scans and Generating Error Maps

The RGB-D camera scans needed to be aligned with the photogrammetry scan in order to allow benchmarking to be performed. Let $X_{i}$ be a [3×N] coordinate vector of the *N* selected points on the RGB-D scan and $Y_{j}$ be the corresponding coordinates of the selected points in the photogrammetry scan. Alignment of the RGB-D scan scans can then be achieved by finding the [3×3] rotation matrix R and the [3×1] translation vector T such that when the RGB-D scan undergoes a ridged body translation the distance between the selected RGB-D and photogrammetry scan points are minimised. This can be expressed as

(1) $$\left[R^{*},T^{*}\right]=\underset{R,T}{argmin}\;\sum_{i,j}\;||Y_{j}-R\;X_{i}-T{\left|\right|}^{2}.$$

Rather than aligning the two scans using manually selected points, the alignment can also be performed automatically using cropped RGB-D and photogrammetry scans and solving Equation (1) using a process referred to as the Iterative Closest Point (ICP) algorithm. Refer to Zinßer et al. [31] for more details on the ICP algorithm used by CloudCompare [32]. The optimised values of R and T can then be used to perform the ridged body translation

(2) $$\bar{X}=R^{*}\;X+T^{*}.$$

on the RGB-D scan to align it with the photogrammetry scan.

**Figure 5:**
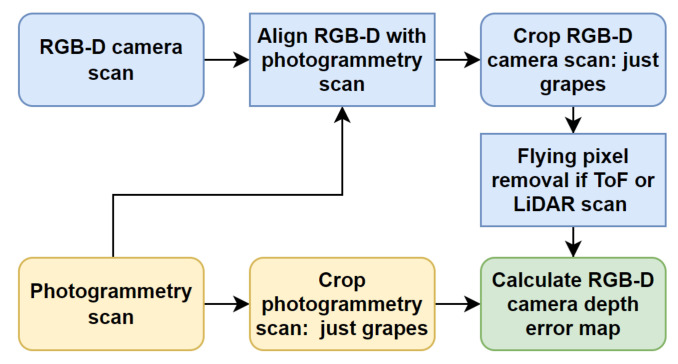
Diagram showing the processing steps used to calculate error depth maps for the RGB-D cameras.

The alignment process described above was initially performed using CloudCompare and manual selection of points on the chequerboard image for both scans. The point clouds were then cropped to just include the grape bunch. An error scan for each RGB-D camera was then obtained. This was calculated by measuring the distance from each point in an RGB-D camera’s scan to the closest point in the photogrammetry scan [33]. Refer to Figure 5 for a block diagram summarising the processing steps used to obtain the depth error maps.

An alternative error analysis method was also used, which aligned the depth camera and photogrammetry scans of the grape bunch using the ICP algorithm, rather than using the chequerboard image. The raw scans were cropped in CloudCompare to just include the scans of the grape bunch. Scaling was also performed on the RGB-D camera scan to correct for projection if this scan was located behind the photogrammetry scan, due to any diffused scattering within the berries. Alignment between the RGB-D scan and the photogrammetry scan was performed using an ICP algorithm. The error in the RGB-D scan was obtained by finding the distance from each point in the ICP aligned RGB-D scan to the closest point in the photogrammetry scan.

#### 2.2.2. Calculating the Proportion of Missing Scan Points

Image processing was performed to estimate the proportion of the scan that was missing for each depth camera relative to the photogrammetry scan. CloudCompare was used to capture 2D images of each depth camera’s scan of the grapes with a white background. To ensure consistency between cameras, these images were obtained using the same viewing angle and position and image size. The percentage of pixels in this image that was white (not grapes) was then calculated using MatLab for each depth camera. The percentage of missing scan area was then obtained by subtracting this value from that obtained for the photogrammetry scan.

#### 2.2.3. Identifying Individual Grapes Using RANSAC

Work was also performed to investigate the potential of identifying and sizing individual grapes from the RGB-D camera scans. The RANSAC algorithm was chosen as it is the method that has been used in the literature related to identifying the position and size of grapes from high-resolution scans captured using commercial scanners. This algorithm fits shapes such as spheres to the scan. Ideally, the size and position of each grape can be identified from the size and position of the corresponding fitted sphere.

CloudCompare was used to apply the RANSAC algorithm to the indoor scans obtained using both the RGB-D cameras and photogrammetry. Schnabel et al. [34] provides a description of the RANSAC algorithm used by CloudCompare [35]. It fitted spheres to the grape bunch scans and used this to segment the scans into a single point cloud for each fitted sphere. Ideally, each of these segmented point clouds would correspond to a different grape. These point clouds were then exported as separate files with the sphere radius in the file name. However, it did not contain the location of the sphere’s centroid.

MatLab was then used to process these segmented point clouds using the least-squares sphere fitting function provided in [36]. For a given camera, each file was loaded and a least-squares fitting of a sphere to the segmented scan was performed to obtain the position of the sphere’s centroid. The closest sphere in the photogrammetry scan was then identified using a K-Nearest Neighbours (KNN) search.

The difference in the 2D position of the RGB-D camera’s sphere relative to the corresponding sphere for the photogrammetry scan was then calculated. This distance calculation did not include an offset in the depth axis direction. This was done to avoid this measurement being dominated by any distance bias that might be present for the depth cameras. Similarly, the difference in the RGB-D camera’s fitted sphere radius relative to the corresponding photogrammetry sphere was also calculated. This process was repeated for all the segmented point clouds and median values obtained. Note that the median was used rather than the average since several fitted spheres were too large relative to the size of the grapes and would have distorted the averaged results. Spheres with a radius greater than 20 mm were ignored when counting the number of fitted spheres.

## 3. Results

**Figure 6:**
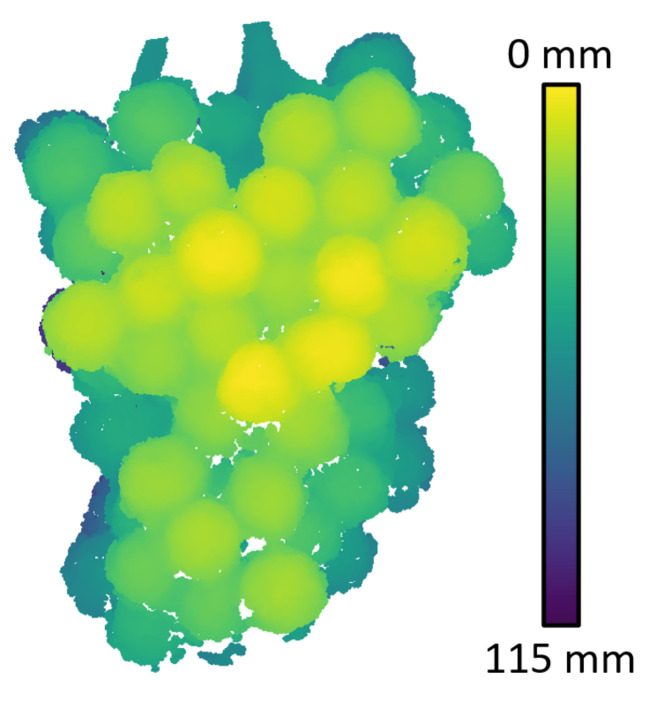
Example photogrammetry 3D depth scan of the grape bunch which was located indoors.

Photogrammetry point clouds of the grape bunches were obtained to act as baseline scans which could be used to evaluate the accuracy of the RGB-D camera scans. Figure 6 provides an example of a high-resolution scan obtained using photogrammetry of the grape bunch for the indoor scans. This scan was obtained using RGB images captured by the Sony A6300 camera. Note that the depth colour scale is relative to the minimum and maximum depth value and has been normalised so that the closest point on the grapes is set to 0 mm. This allows comparisons of depth maps to be made across cameras.

**Figure 7:**
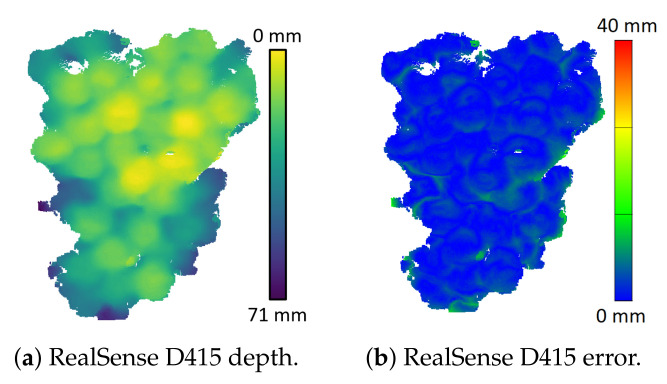

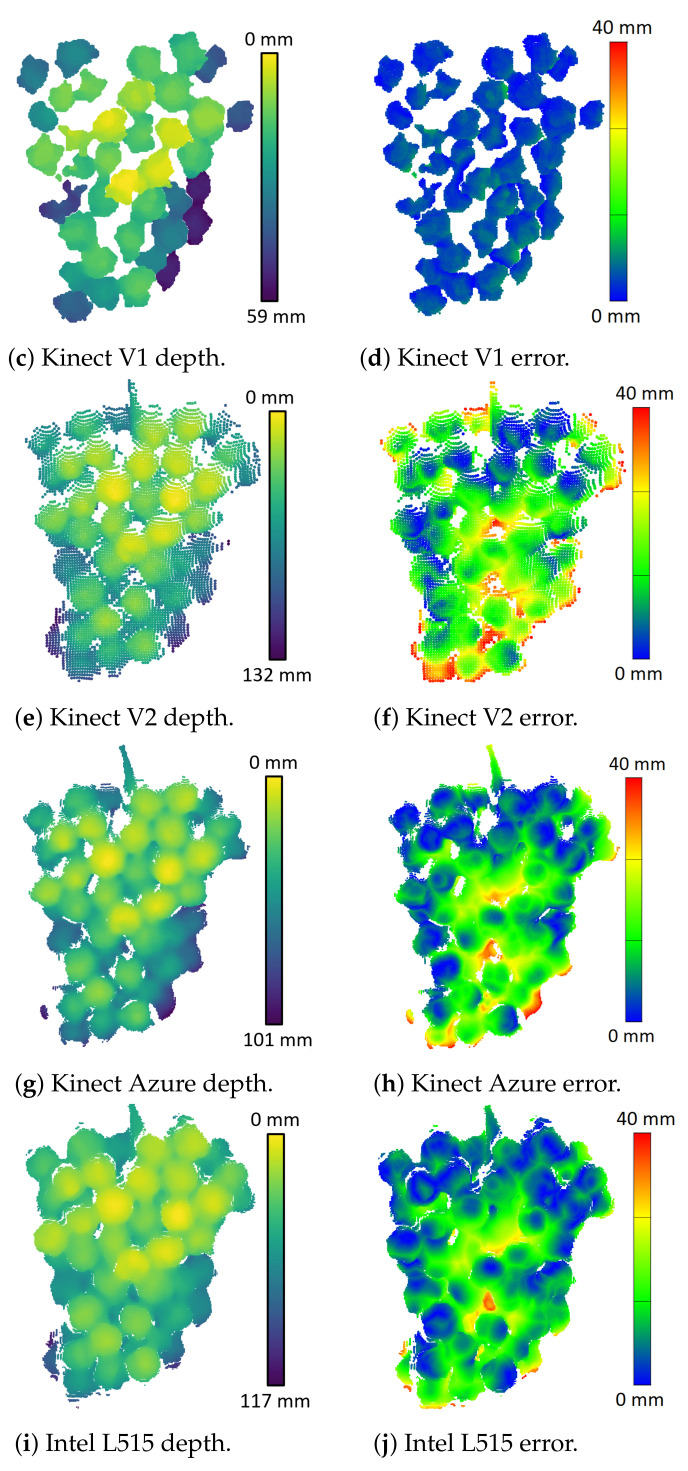
Depth and error scans (relative to the photogrammetry scans) for the RGB-D cameras located indoors at a distance of 600 mm from the unpainted grape bunch. An error bar is provided that shows the colour scale for the error scans and is the same for all the cameras. The colours for the depth scans are relative to the maximum and minimum depth of the point cloud for each camera.

The photogrammetry scan was used as a ground truth to obtain error maps for depth scans captured by the RGB-D cameras. Figure 7 shows the depth and error scans of the RGB-D cameras, which were captured indoors with the cameras located at a distance of 600 mm from the grapes. Note that these error scans were obtained by aligning the depth camera and photogrammetry scans using the chequerboard image and not using the ICP alignment method. These results show that the ToF and LiDAR cameras give depth scans of the grape clusters that had distances biased to be further away than they should be. This effect was not observed for the Kinect V1 or the RealSense D415 cameras. It was believed that diffused scattering in the grapes could be the cause of the distance bias for the ToF and LiDAR cameras. The following section investigates this further.

### 3.1. Investigation of Distortion Effects

**Figure 8:**
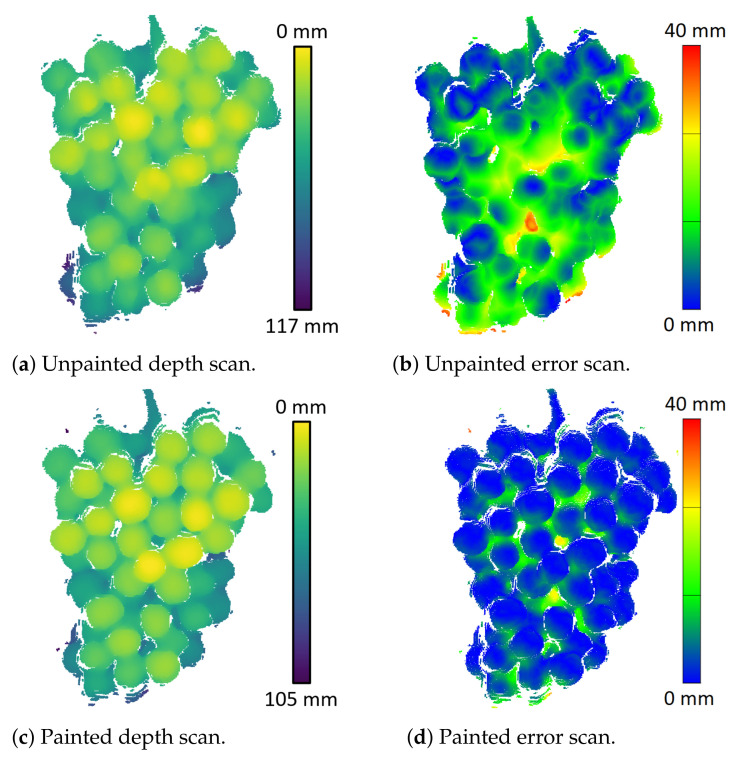
Depth and error scans for the Intel L515 before and after spray painting the grape bunch with white paint.

The grapes were spray-painted with white paint to investigate if diffused scattering was causing the distance bias for ToF and LiDAR cameras. Figure 8 provides examples of the Intel L515 LiDAR depth scans for a grape bunch before and after it had been sprayed with paint. The painted scans have the depth error bias removed and the clarity of individual berries in the depth map appears to be slightly enhanced.

Table 2 provides the mean distance error for the grapes bunch for scans made before and after the grapes were spray-painted. No significant difference in the error (only 0.5 mm) was observed between the unpainted and painted scans for the Kinect V1 and RealSense D415, which are SL and AIRS cameras. However, we can see that painting the grapes reduces the distance bias for the ToF and LiDAR cameras.

ICP alignment error analysis was also performed. This method appears able to remove the distance bias in post-processing, see the third column of Table 2. However, the errors for the ToF and LIDAR cameras are still slightly higher than their SL and AIRS counterparts.

**Figure 9:**
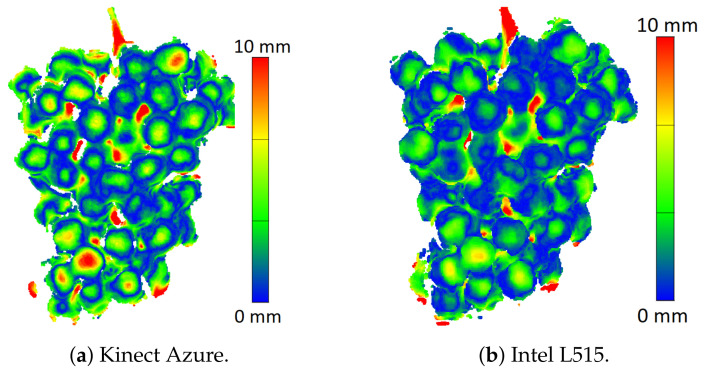
Kinect Azure and Intel L515 error scans for unpainted grapes after the depth camera scans were aligned with the photogrammetry reference scan using the ICP alignment method.

Figure 9 shows the error maps for the Kinect Azure and Intel L515 cameras where ICP had been used to align their RGB-D depth scans with the photogrammetry scans. While this appears to have removed the distance bias, it shows that shape distortion errors still occur in the form of peaks located at the centre of each individual grape. The ToF cameras appeared to show slightly more pronounced shape distortions compared to the LiDAR.

**Figure 10:**
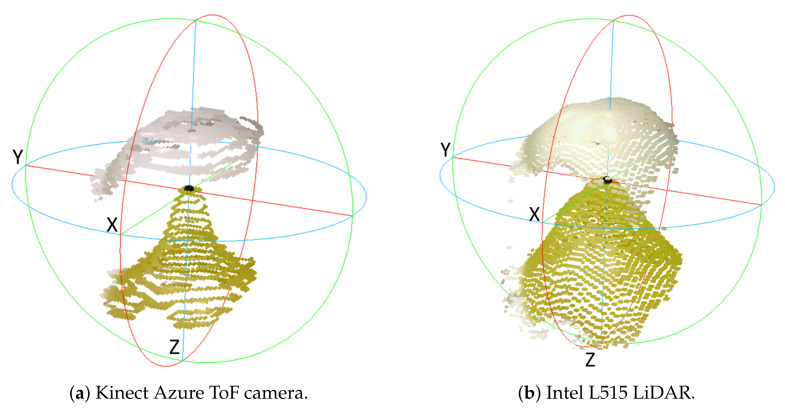
Scans for Kinect Azure (**a**) and Intel L515 (**b**) RGB-D depth scans of a single grape before (green) and after (white) individual grapes had been painted with white spray paint. Note that the *Z*-axis direction shown in the plots is the depth axis. The cameras were located 350 mm from the grapes. The Kinect Azure and Intel L515 have their unpainted peaks respectively about 7 mm and 8.5 mm behind the painted peaks. The Azure scan is more heavily quantised than the L515 scan.

**Figure 11:**
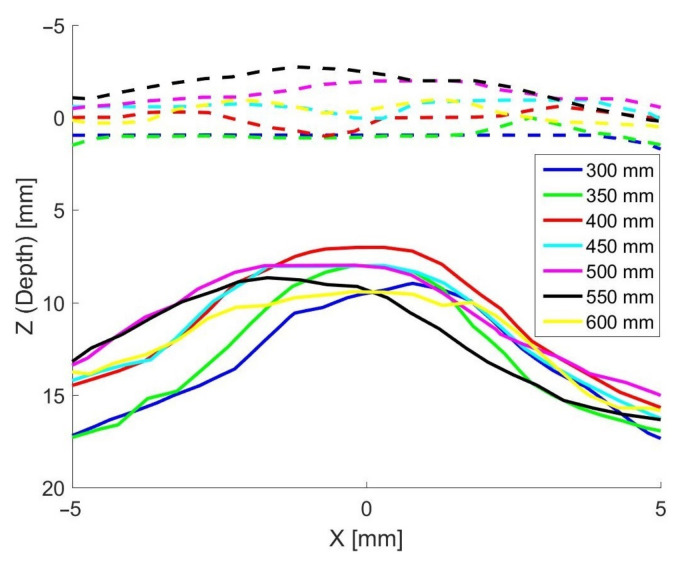
Plots showing cross-sections of scans made by the Kinect Azure of a single grape before (solid lines) and after (dashed lines) the grape had been painted. The different colours represent scans made with the camera being located at distances from the grape ranging from 350 to 600 mm.

This distortion effect is illustrated in Figure 10. This plot shows scans captured by the Kinect Azure and Intel L515 of this grape before and after it was painted. These RGB-D cameras were located at a distance of 350 mm from the grapes. This distance was chosen as the distortion appeared slightly more pronounced at this distance, as is illustrated in Figure 11. The unpainted grape scan points show significantly more pointed shape distortion compared with the painted grape.

Figure 11 shows cross-sections in the *X*-*Z* plane of Kinect Azure scans made of a single grape before and after it had been sprayed with paint, for a range of distances of the depth camera from the grape. The depth has been normalised so that zero depth corresponds to the front of the ring supporting the grape. The distance bias and shape distortion are reduced when the grape is painted. It appears that the shape distortion is more pronounced when the camera is closer to the grape.

**Figure 12:**
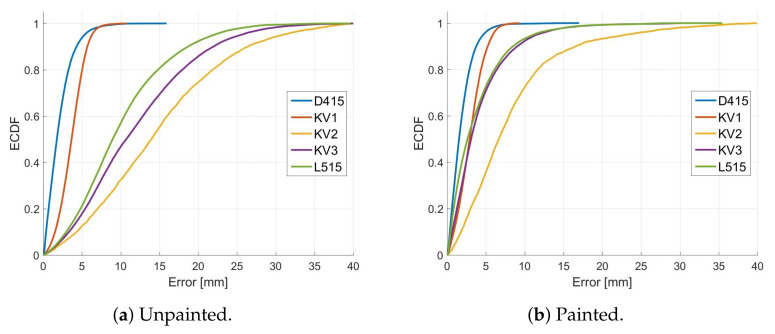
Plots (**a**,**b**) respectively show the ECDF error measurements for the grape bunch scans made indoors before and after the grapes had been sprayed with paint. The cameras were positioned 600 mm from the grapes.

Figure 12 provides plots of the Empirical Cumulative Distribution Functions (ECDF) of the errors in scans captured indoors both before and after the grape bunch had been sprayed with white paint. The ECDF plots show what percentage of the errors is below a given value. For example, we can see that, for the unpainted grapes, the Kinect V2 has 95% of its errors below 30 mm. In contrast, the corresponding scans for the RealSense D415 has 95% of its errors less than about 5 mm.

Note that some caution is required when interpreting the ECDF plots. This error analysis only looks at errors in scan points captured with the depth cameras. However, it does not analyse how much of the scan was missing. For example, the ECDF plot shown in Figure 12 indicates that the Kinect V1 produced relatively low errors. However, from Figure 7 we can see that there was a significant proportion (about 20%) of the scan that was missing compared with the other cameras. Additionally, the ECDF does not provide information on how well individual grapes can be identified within a scan.

### 3.2. Measurements Made in Direct Sunlight

Measurements were also made using the cameras located outdoors to evaluate their performance in direct sunlight. Note that the grapes used for the indoor scans had been painted in order to investigate how diffused scattering within the berries affected the results. Hence, a different grape bunch was used for the outdoor scans. However, the methodology was designed with the aim of providing results that were independent of which grape bunch was used in the benchmarking by comparing the photogrammetry and RGB-D camera scans. This means that the error analysis should be relatively independent of the grape bunch used, though some difference in the results may occur.

**Figure 13:**
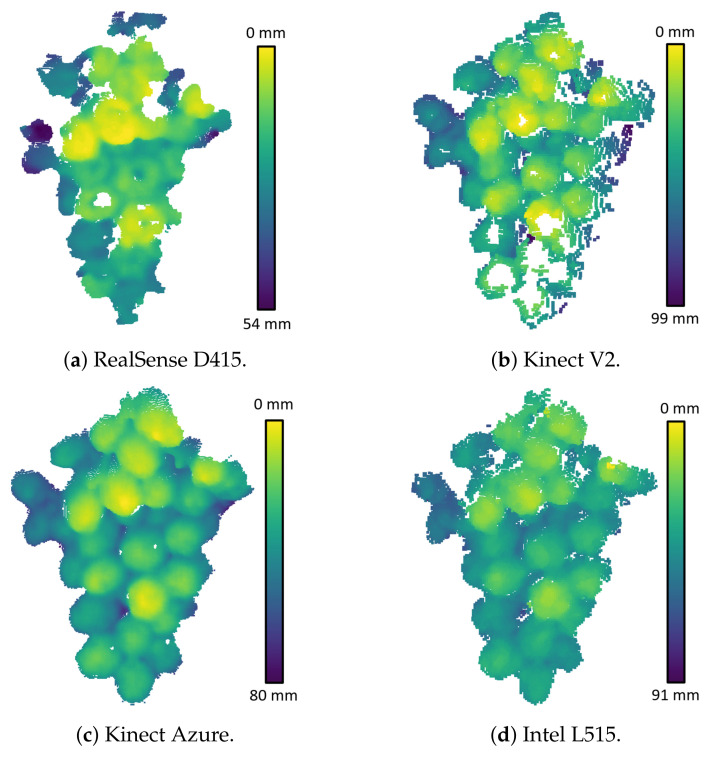
Depth scans for the RGB-D cameras captured outdoors at a distance of 600 mm from the grape bunch.

Figure 13 shows examples of these depth scans with the cameras at a distance of 600 mm from the grapes. Note that no results are shown here for the Kinect V1. This is because no measurements were able to be achieved with this camera until after sunset. All of the other depth cameras were able to obtain scans of the grapes in direct sunlight. However, the errors for the RealSense 415 are similar to those of the Kinect V3 and LiDAR for outdoor measurements but are still lower than those for the Kinect V2.

**Figure 14:**
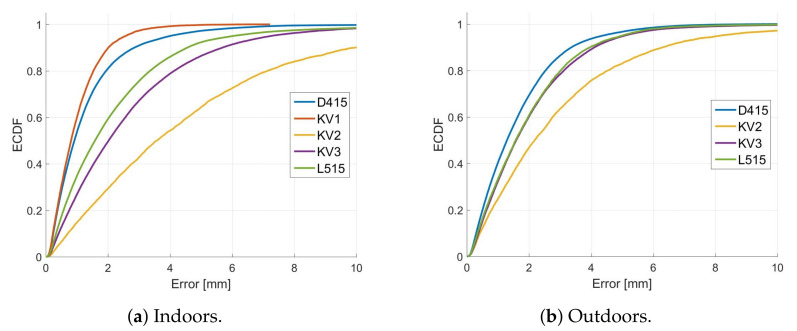
Plots comparing ECDF plots for scans of two different unpainted grape bunches which were captured by depth cameras (**a**) indoors and (**b**) outdoors in direct sunlight using ICP alignment of the depth camera scans with the photogrammetry scans. The grapes were located 600 mm from the cameras.

Figure 14 compares ECDF plots for these scans made outdoors with the scans made indoors where ICP alignment has been used. Table 3 provides a comparison of the proportion of missing scan points for each camera for both indoor and outdoor measurements. It can be seen that the RealSense D415 has a 13% increase in the proportion of missing scan points for outdoor measurements, while the ToF and LiDAR cameras are relatively unaffected. There is a slight (2%) reduction in the proportion of missing scan points for the Kinect V2 outdoors relative to indoors. However, this is probably within the measurement error for this analysis method or may be due to the fact that different grape bunches were used for the indoor and outdoor experiments.

### 3.3. Detection of Individual Grapes Using RANSAC

**Figure 15:**
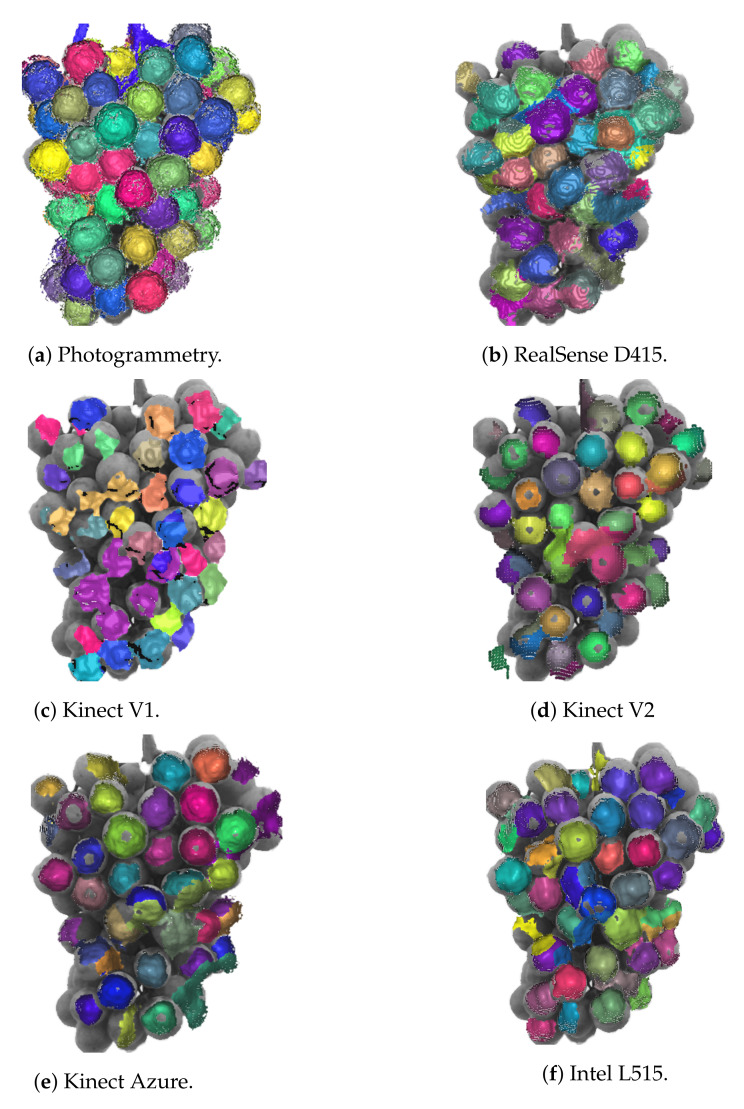
Plots showing the output of the RANSAC algorithm on the grape depth scans which were captured indoors. This is overlaid over a greyscale photo of the grape bunch for reference.

Analysis was performed on the grape scans that were captured indoors to investigate if it was possible to detect and size individual grapes from the raw RGB-D camera depth scans. The RANSAC algorithm within CloudCompare was used to fit spheres to the depth scans. Figure 15 shows the resulting segmentation of the scans provided by the RANSAC sphere fitting for the photogrammetry and depth camera scans. These are overlaid over a photo of the grapes for comparison. The different colours correspond to different segmented point clouds obtained by fitting spheres to the raw scans. Ideally, there would be a separate colour for each grape. However, it can be seen that the results are not perfect. The performance of the algorithm is lower for the RGB-D cameras scans compared to that of the photogrammetry scan.

Table 4 provides the median difference in the detected 2D position and sphere radius relative to the corresponding spheres for the photogrammetry scans. The medium sphere radius for the photogrammetry scans was 13.7 mm. The depth information was ignored when calculating the 2D position error since adding depth would have resulted in values that were dominated by the distance bias for the ToF and LiDAR cameras. The median differences in the 2D positions of the spheres are relatively low. These position errors may be related to errors in the alignment of the depth camera scans in comparison to the photogrammetry scan.

This table also gives the number of spheres detected for each RGB-D camera that had radius values less than 20 mm. We can also see that the ToF and LiDAR camera scans have smaller median sphere radius values compared to those obtained using photogrammetry and the RealSense D415 and Kinect V1 cameras.

## 4. Discussion

The RealSense D415, which uses AIRS technology, was the most accurate camera indoors. However, it showed reduced performance outdoors. This is in line with the findings of Kurtser et al. [26] that reported increased errors for the RealSense D435 AIRS camera with increased sunlight exposure. The ECDF plots shown in Figure 14 indicate that the errors for the RealSense D415 increased outdoors but were still similar to that of the Kinect Azure and Intel L515 (after correcting for their distance bias using ICP). However, the RealSense D415 also had a significant increase in missing scan points when operated in direct sunlight. This is illustrated in Table 3, where the percentage of missing scan points relative to the photogrammetry scan increased from about 1% to 14% when measurements were made outdoors. Additionally, the 3D shape of individual grapes was less pronounced, which would make it harder to identify and measure the size of the grapes. This might be because it was not able to use its projected IR pattern due to saturation by sunlight. Saturation of the stereo IR cameras may also have occurred. Moreover, the camera may have struggled with the dynamic range caused by direct illumination from the sun with shadows.

The Kinect V1 SL camera also had low depth errors for measurements made indoors. However, Table 3 shows that it had about 20% of the scan points missing, which was the highest of any of the other cameras. This resulted in a smooth shaped scan of the grape bunch and did not display the valleys between grapes. This phenomenon can be seen in the plots presented by Marinello et al. and Hacking et al. [21,22,23]. The Kinect V1 has a significant deterioration in resolution as the distance of the grapes from the camera increases, as reported by Marinello et al. [21]. This appears to be related to the strong depth quantisation dependence on scan depth for this camera.

The Kinect V1 could not be used for scanning grapes outdoors in direct sunlight. This was expected since its projected IR pattern would have been saturated by the sunlight. Hacking et al. [22,23] had also reported issues with its performance when used outdoors. They had therefore suggested that the Kinect V2 should be investigated for outdoor grape bunch scanning since it would be more robust to sunlight.

The cameras that used ToF technologies were found to be more robust to sunlight conditions. Both the Kinect Azure and Intel L515 appeared to provide similar results indoors and outdoors in direct sunlight. The Kinect V2 had higher errors than the Azure and Intel L515. It was able to operate in sunlight but did have some issues with saturation resulting in scan points being missing. This may be addressed by adjusting the exposure in software.

The ToF and LiDAR cameras produced scans of the grapes that had a distance bias of about 8 mm and had a distortion in the shape of the scans of the grapes, which was not observed for the SL and AIRS cameras. The shape distortion for the ToF and LiDAR cameras makes individual grapes within the scan more prominent and easier to identify than the Kinect V1 and the RealSense D415. This distortion may therefore be beneficial for counting individual grapes. The plots in Figure 8, Figure 10 and Figure 12 show that these distortion effects were largely removed when the grapes were painted. This indicates that the distance bias and shape distortions are due to diffused scattering within the berries of the transmitted light used by these cameras.

The Intel L515 LiDAR appeared to have slightly less distance bias and distortion compared to the two ToF cameras. The difference in distortion between the ToF and LiDAR cameras may be due to the process they used to emit light. ToF cameras emit light using a single wide-angle coded pulse and captures the returning light from a range of locations simultaneously as pixels. If this light pulse enters a grape and experiences diffused scattering, each pixel of the ToF camera corresponding to the grape will receive some combination of light entering across the entire surface of the grape visible to the camera. In contrast, LiDARs typically build up the point cloud in a scanning process making measurements at a single scan point location at a time. This means that the light detected by the LiDAR may be more localised within the grape compared with the ToF camera. Given the different methods used by the two types of cameras, it is perhaps understandable then that each would have a different distortion pattern.

There have been a few reports of ToF cameras having a distance bias in fruit due to diffused scattering. Neupane et al. [37,38] reported that ToF cameras provided distance measurements for mangoes, which were biased to be slightly too large, due to diffused scattering within the fruit. This distance bias increased over several days and was suggested as a means of measuring the ripeness of the mango fruit. Sarkar et al. [39] used this phenomenon to investigate the ripeness of apples using a ToF camera and polarisers. However, we have not seen any previous report of a shape distortion in ToF camera scans of fruit. The fact that the shape distortion is so pronounced for grapes may be due to the comparatively smaller size of the berries and relatively higher translucent properties compared to the other fruit that has been investigated previously.

This raises the question, could the distortion of RGB-D cameras that use ToF technology be used to provide a non-destructive estimation of grape properties such as ripeness? Future work is planned to investigate how the distortion effects vary with berry ripeness and size. This might also give some insight into the potential of correcting the ToF and LiDAR scans for these distortions in post-processing.

The ability to identify individual grapes from 3D scans could be beneficial. It potentially could allow the number and size of berries in bunches to be measured. Additionally, it might allow more accurate yield estimation through 3D bunch architecture modelling. There have been several works that have used RANSAC to detect and size grapes. However, these works used high-resolution 3D scans captured using commercial laser and structured light scanners [5,7,8,9,10] and using photogrammetry [13], not depth cameras. Yin et al. [29] used RANSAC to fit cylinder shapes to the ZED RGB-D camera scans of grape bunches. However, this was related to the pose estimation of the entire grape bunch for robotic harvesting applications and did not attempt to fit individual grapes.

The RANSAC algorithm was used in this work on both the photogrammetry and RGB-D camera scans. The RANSAC algorithm showed some promise for detecting individual grapes in the RGB-D camera scans. All of the RGB-D cameras gave similar median 2D positions for the spheres/grapes relative to photogrammetry, as indicated in Table 4. However, the RANSAC algorithm produced fitted spheres with a smaller radius for the ToF and LiDAR cameras. This was to be expected given the shape distortion observed for these cameras.

The ability of RANSAC to correctly segment out individual berries was lower for the RGB-D cameras compared with that for the photogrammetry scans. As an example, in Figure 15, it can be seen that the Kinect V1 shows multiple grapes close to each other that have the same colour. This indicates that the algorithm has failed to separate these particular berries out as separate spheres. In contrast, a much higher proportion of the berries are correctly segmented for the photogrammetry scan.

The RANSAC algorithm also identified more grapes in the photogrammetry scans compared to that in the RGB-D camera. This is particularly pronounced for the grapes located around the edges of the bunch. However, this would appear to be mainly related to the way the photogrammetry scans are obtained using images captured from a range of positions relative to the grape bunch. The RGB-D camera images shown here in contrast are captured from a single location. This means the RGB-D cameras see a lower proportion of the surface area of the grape bunch. Improved results could be obtained by merging multiple RGB-D camera scans taken at a range of positions and angles relative to the grapes. This could be achieved using SLAM or a similar point cloud alignment technique [14]. This should then make the RGB-D camera scans more comparable to the photogrammetry scans.

### Future Work

More investigation is needed to ascertain the optimal method of detection and sizing the grapes from RGB-D camera scans. Future work could look at fitting other shapes to the grape scans such as ellipsoids or a shape that is similar to the distortions due to diffused scattering effects for the ToF and LiDAR cameras. Additionally, custom-designed algorithms may be needed for these cameras. This may include correction of the distortion effects for these cameras.

The ToF and LiDAR cameras had slightly higher errors compared with the other two cameras indoors even when the grapes were painted or when the distance bias had been removed in post-processing. It is possible that these errors could be reduced if additional filtering of the flying pixels was performed. However, this could potentially result in removing real scan points partially in the valleys between individual grapes. It is also possible that the error analysis process used here is overestimating the errors slightly for these cameras.

Improvements in the error analysis technique used in this work could also be performed. The error in the RGB-D cameras scans was obtained by comparing their depth scans with those obtained using photogrammetry. There could be some small errors in these photogrammetry scans. It appears that these scans had some smoothing in the valleys between grapes in a similar manner to the RealSense D415. It would be interesting in future work to use an alternative scanning system such as a commercial laser scanner for obtaining the ground truth scans.

The method used to calculate the distance errors could be improved in future work, particularly for the scans where a distance bias is present. One option could be to project a line from the location of the RGB-D camera to a scan point in its depth scan. One could then calculate the point on the line which is closest to a scan point on the photogrammetry scan (or where it passes through a mesh surface obtained from the photogrammetry scan). The distance along the line from that point to the RGB-D scan point could then be used as the depth error.

This work was performed with green grapes. Some preliminary testing with red grapes indicated that these also had a shape distortion and distance bias that appeared similar to that observed in the green grapes. However, this was not investigated in detail and more work is needed with other types of grapes.

The measurements described in this work were performed in controlled lab type environments. This was appropriate for the type of investigations performed in this study. However, it should be noted that achieving a fully automated system in a real vineyard environment would be more challenging. For example, this would require segmentation to allow automatic identification of grapes from leaves and stems [27]. There may also be occlusions by leaves or other grape bunches. More work is needed to address these types of challenges.

## 5. Conclusions

The Kinect V1 is no longer in production and hence is unlikely to be used in the future for grape yield estimation. However, it provides a comparison of the IR structure light technology with that used by other RGB-D cameras. The Kinect V1 was not able to function in direct sunlight. This is likely to be due to its projected IR pattern being saturated by sunlight. This indicates that RGB-D cameras that operate using IR structured light would only be suitable for measurements made at night or with a cover system that blocks out sunlight.

The Kinect V1 provided scans made indoors (out of direct sunlight) with relatively low errors for the parts of the grapes facing the camera. However, it did not capture portions of the grapes, particularly in the valleys between individual grapes. While this might be adequate for rough volume estimations using a convex hull or mesh of the grape bunch scan, it does make identifying and sizing of individual grapes within the scan difficult. This is illustrated in the RANSAC results where the segmentation process struggled to correctly separate out many neighbouring grapes. In addition, it appears that the depth scans for the Kinect V1 had a relatively high quantisation compared with the other cameras.

The RealSense D415, which uses active stereoscopy, provided the lowest errors of the cameras analysed. Its indoor scans did not have the missing scan points or quantisation that was seen in the Kinect V1. However, it smoothed out the valleys between the grapes making it harder to detect individual grapes from the depth scans. The scans made with this camera in direct sunlight had slightly higher errors and missing scan points. In future work, we would look at adjusting the exposure of this camera in software to see if this issue can be addressed. However, it appears that sunlight was saturating its projected IR pattern, meaning it was acting purely as a passive stereo camera. This might indicate that cameras that operate using the AIRS technology may not have any additional benefit for yield estimation made in sunlight conditions compared with RGB-D cameras which operate using just passive stereo technologies. This may be investigated in future work.

The ToF (Kinect V2 and Kinect Azure) and LiDAR (Intel L515) cameras provided the best ability to detect individual grapes compared to the other cameras. However, they produced 3D scans of the grapes which were biased to give depth distances that were too large. Additionally, these cameras also produced distortions in the scans in the form of peaks centred on each grape location.

The distance bias and shape distortion were removed when the grapes were painted. This indicated that the distance bias and distortion were the results of diffused scattering within the grape. Previous work such as Neupane et al. [37] had reported measuring a distance bias for fruit using ToF cameras and have related this to the ripeness of the fruit. However, we are not aware of any previous studies which have reported a distortion in the shape of the scans of the fruit. It may be that this distortion is enhanced due to the small size of grape berries and their translucent properties.

The distance bias found in the LiDAR and ToF cameras scans of the grapes may not be an issue if one is only interested in the shape of the grape bunch. In fact, the distortion pattern makes it easier to identify individual grapes compared with the SL or AIRS cameras. However, more work is needed to investigate how much this distance bias and distortion affect the accuracy of grape volume/yield estimations. In our study, it did result in smaller detected berry diameters obtained using RANSAC compared with the other cameras. More work is needed to understand what factors such as ripeness, berry size, and variety play in the magnitude of the distance bias and shape of the distortion. With more understanding of these factors, it may be possible to use these distortions to perform non-destructive measurement of grape properties such as ripeness or possibly to correct for the distortions in post-processing.

In future work, we plan to investigate further the potential of the ToF and LiDAR cameras since they were less affected by sunlight and there is potential to utilise the distortion present in their scans for more accurately identifying individual berries. Additionally, there may be opportunities for using the distortion for non-destructive testing of berry properties.

## Figures and Tables

**Table 1:** List of RGB-D cameras used with the depth measurement technologies they use and their resolution and field of view specifications.

Camera	Technology	Resolution [Pixels]	Field of View [Deg]
RealSense D415	AIRS	1280 × 720	65 × 40
Kinect V1	SL	640 × 480	57 × 43
Kinect V2	ToF	512 × 424	70 × 60
Kinect Azure	ToF	1024 × 1024	120 × 120
Intel L515	LiDAR	1024 × 768	70 × 55

**Table 2:** Mean depth error for RGB-D camera scans of the grapes before and after they had been sprayed with paint. The cameras were located indoors and were positioned 600 mm from the grapes.

Camera	Unpainted [mm]	Painted [mm]	Unpainted with ICP Alignment [mm]
RealSense D415	2.13	1.88	1.33
Kinect V1	3.67	3.00	1.01
Kinect V2	14.7	8.28	4.73
Kinect Azure	11.9	4.19	2.66
Intel L515	10.0	3.82	2.17

**Table 3:** Estimate of the percentage of the depth scan that is missing for each camera relative to that obtained using the photogrammetry scans.

Camera	Indoors [%]	Outdoors [%]
RealSense D415	0.9	14
Kinect V1	20	–
Kinect V2	14	12
Kinect Azure	4.2	4.6
Intel L515	2.0	3.6

**Table 4:** Information on the RANSAC algorithm fitting of spheres to individual grapes in the scans. This shows the number of spheres detected and the median difference in the radius and 2D positions of the spheres for the RGB-D cameras relative to the same spheres in the photogrammetry scans.

	RealSense D415	Kinect V1	Kinect V2	Kinect Azure	Intel L515
**No. of Spheres Detected**	25	22	31	26	30
**Median Radius Difference [mm]**	1.7	2.8	−3.7	−3.5	−3.0
**Median Position Difference [mm]**	1.9	2.1	3.5	2.0	2.1

## Data Availability

Not applicable.
